# Supplementary material for: Overexpression of the Aspergillus fumigatus Small GTPase, RsrA, Promotes Polarity Establishment during Germination
Source: J Fungi (Basel). 2020 Nov 13;6(4):285. doi: 10.3390/jof6040285 (PMC7711769; doi:10.3390/jof6040285)
Supplement: Supplementary file 1 [file jof-06-00285-s001.zip › Supplemental figures/Fig. S1.pptx]

## Slide 1
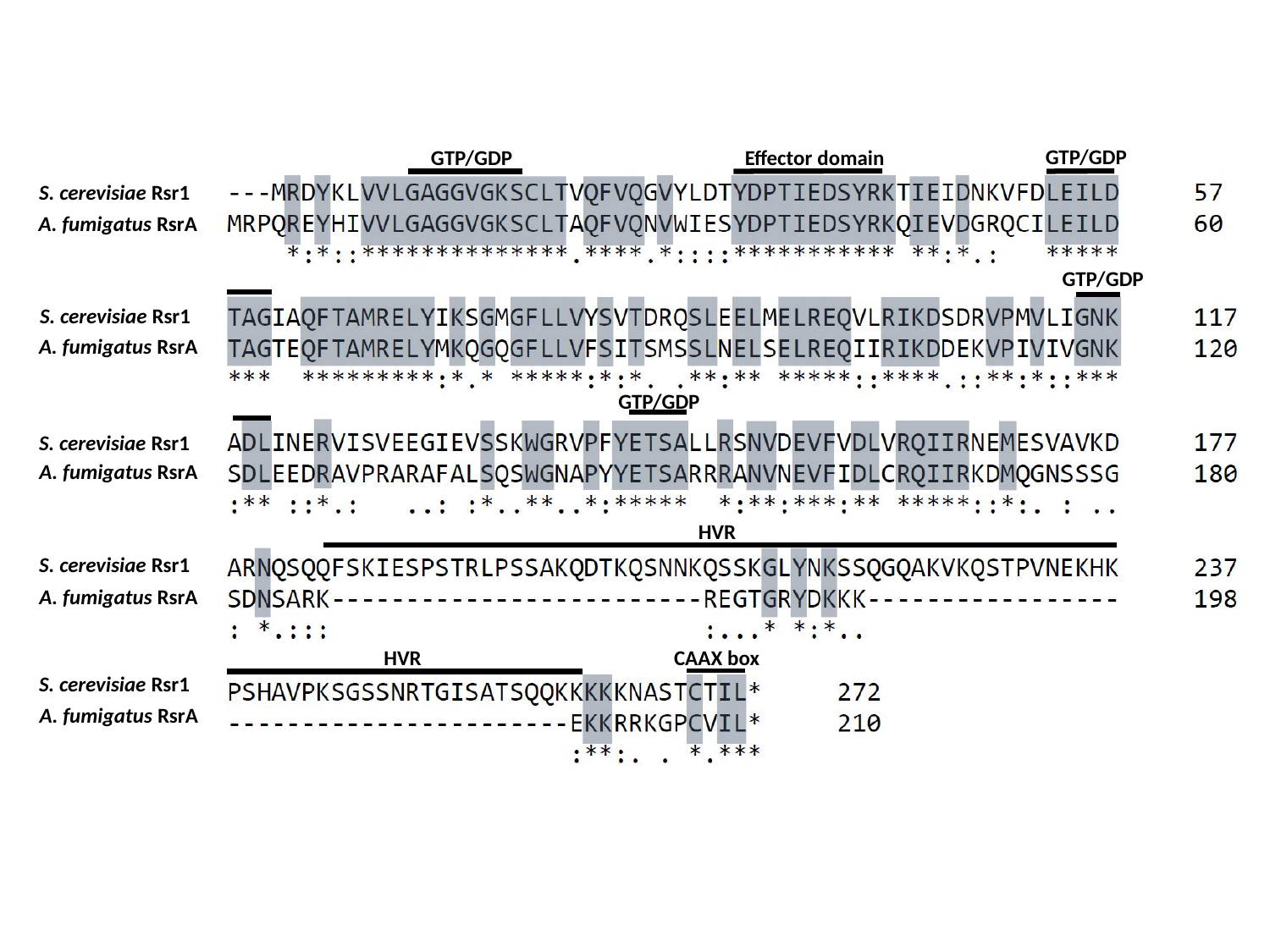

GTP/GDP
Effector domain
GTP/GDP
S. cerevisiae Rsr1
A. fumigatus RsrA
GTP/GDP
S. cerevisiae Rsr1
A. fumigatus RsrA
GTP/GDP
S. cerevisiae Rsr1
A. fumigatus RsrA
HVR
S. cerevisiae Rsr1
A. fumigatus RsrA
HVR
CAAX box
S. cerevisiae Rsr1
A. fumigatus RsrA
