# Supplementary material for: Overexpression of the Aspergillus fumigatus Small GTPase, RsrA, Promotes Polarity Establishment during Germination
Source: J Fungi (Basel). 2020 Nov 13;6(4):285. doi: 10.3390/jof6040285 (PMC7711769; doi:10.3390/jof6040285)
Supplement: Supplementary file 1 [file jof-06-00285-s001.zip › Supplemental figures/Fig. S2.pptx]

## Slide 1
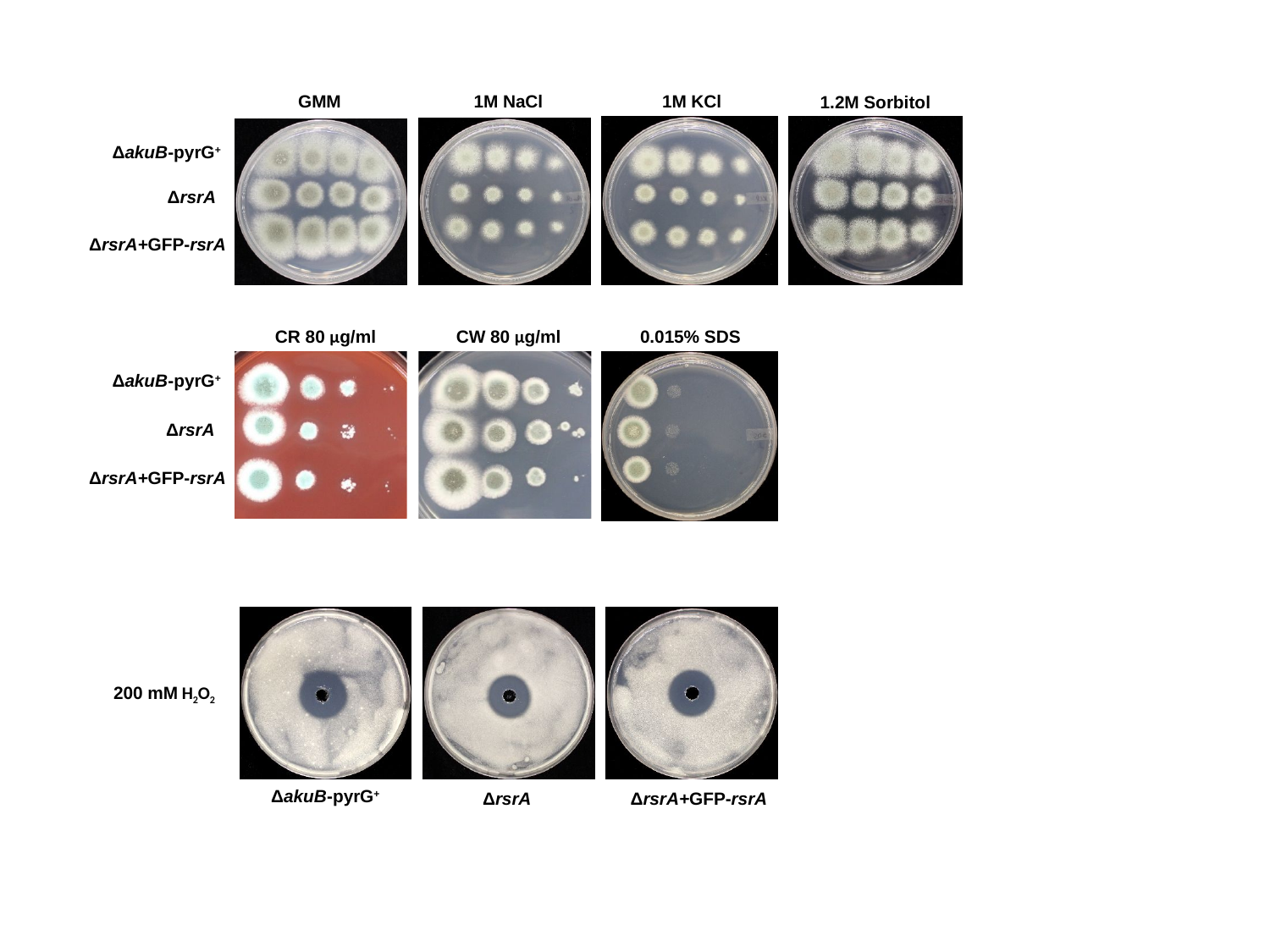

1M NaCl
1M KCl
GMM
1.2M Sorbitol
ΔakuB-pyrG+
ΔrsrA
ΔrsrA+GFP-rsrA
CR 80 μg/ml
0.015% SDS
CW 80 μg/ml
ΔakuB-pyrG+
ΔrsrA
ΔrsrA+GFP-rsrA
200 mM H2O2
ΔakuB-pyrG+
ΔrsrA
ΔrsrA+GFP-rsrA
