# Supplementary figures and images for: Overexpression of the Aspergillus fumigatus Small GTPase, RsrA, Promotes Polarity Establishment during Germination
Source: J Fungi (Basel). 2020 Nov 13;6(4):285. doi: 10.3390/jof6040285 (PMC7711769; doi:10.3390/jof6040285)

## Slide 1
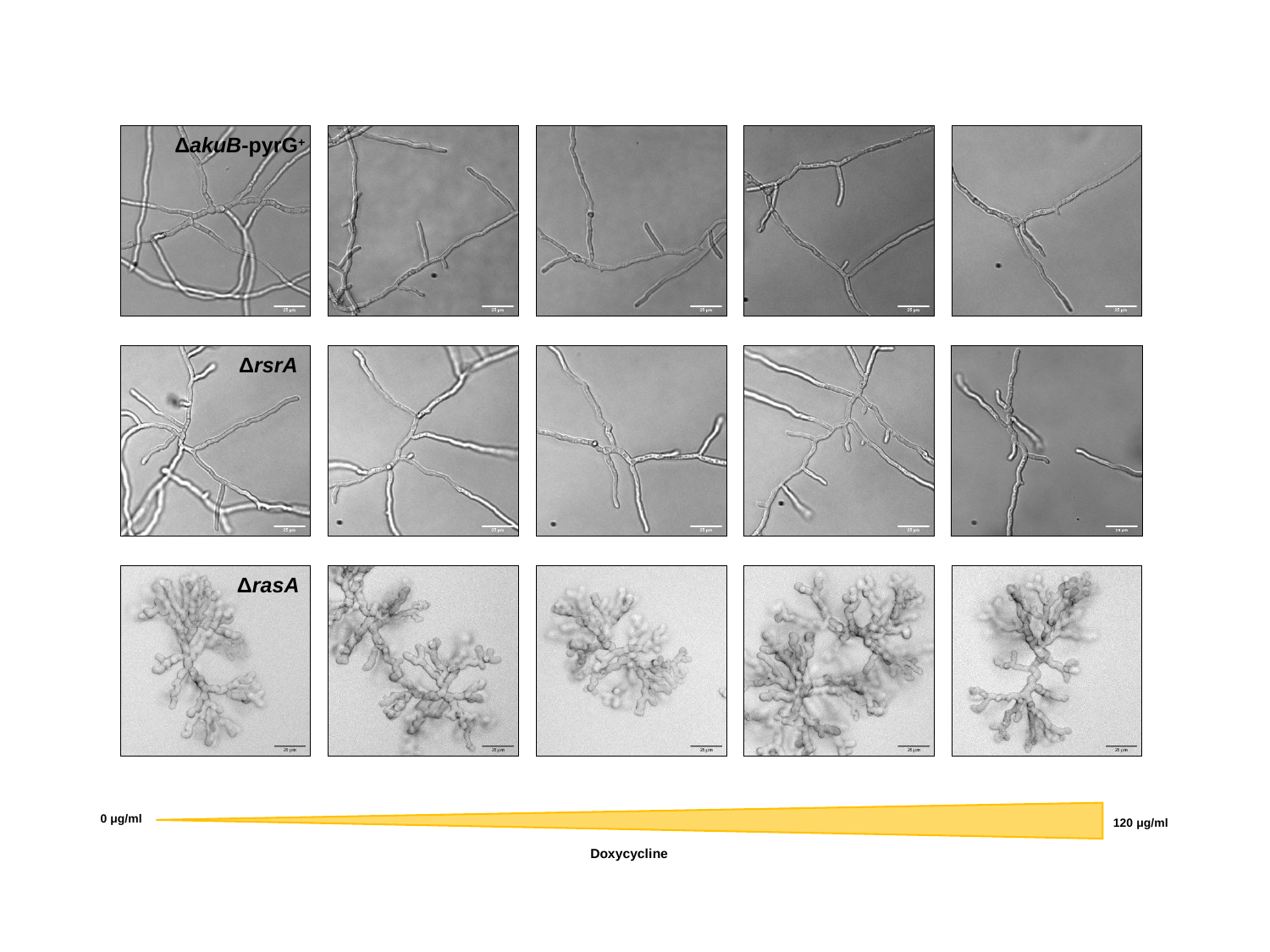

ΔakuB-pyrG+
ΔrsrA
ΔrasA
0 μg/ml
120 μg/ml
Doxycycline

Supplement: Supplementary file 1 [file jof-06-00285-s001.zip › Supplemental figures/Fig. S3.pptx]
